# Delayed aortic arch deformity associated with a frozen elephant trunk after total arch replacement

**DOI:** 10.1186/s13019-026-04576-x

**Published:** 2026-07-28

**Authors:** Jin Kato, Kenichi Hashizume, Hideyuki Shimizu

**Affiliations:** 1 Department of Cardiovascular surgery, Keio University Hospital, Tokyo, Japan; 2 35 Shinanomachi, Shinjuku-ku, Tokyo, 160-8582 Japan

**Keywords:** Aortic dissection, Frozen Elephant Trunk, Computed tomography, Migration

## Abstract

We report a case of progressive deformity of the native distal aortic arch at the level of the tip of a frozen elephant trunk (FET), which developed 5 years after total arch replacement with FET (TAR-FET) for acute Stanford type A aortic dissection. This deformity arose in the late postoperative period and progressed rapidly over the following 6 months without any change in aortic diameter, and no clinical symptoms—including upper-to-lower extremity blood pressure discrepancy—were observed throughout this course. The patient ultimately required re-total arch replacement with FET extension, a concomitant Bentall procedure, and thoracic endovascular aortic repair (TEVAR), the latter performed emergently on postoperative day 1 after detachment of the new FET from the descending aortic wall produced a marked upper-to-lower extremity pressure gradient; this resulted in complete restoration of aortic morphology. This case demonstrates that morphological change of the aortic arch after FET can progress silently in the late postoperative period and may not be detected by diameter-based surveillance criteria. It highlights the importance of evaluating aortic morphology, in addition to diameter, and of selecting an adequately long distal landing zone extending beyond the arch curvature, during long-term surveillance and reintervention planning.

## Introduction

The frozen elephant trunk (FET) technique is widely used for acute Stanford type A aortic dissection and other complex aortic arch pathologies. Late complications, including proximal graft migration, kinking deformity, distal stent-induced new entry (dSINE), and hemodynamic compromise, are well recognized, and additional distal interventions are required in a non-negligible proportion of patients over time [1–3]. Long-term outcome series of total arch replacement with FET (TAR-FET) report reintervention rates affecting a meaningful minority of patients during follow-up [8, 9, 11], and consensus statements increasingly emphasize structured long-term imaging surveillance after FET implantation [6, 7].

Current reporting standards define negative remodeling after dissection repair in terms of diameter change, such as false lumen enlargement, true lumen reduction, or an increase in total aortic diameter [4]. In contrast, comparatively little attention has been paid to morphological deformity of the aortic arch in the absence of diameter change. Studies describing patterns of aortic remodeling after TAR-FET have noted that morphological configuration, and not diameter alone, can evolve over time [10, 12], and emerging evidence suggests that arch morphological changes after FET may be associated with late aortic events independently of aortic diameter [5, 20], but clinical descriptions of this trajectory remain limited.

We encountered a case in which arch deformity progressed over 5 years of follow-up without diameter change and in the complete absence of symptoms, then worsened rapidly over the following 6 months. We report this case as an educational example of a pattern of deterioration that may not be captured by current diameter-based surveillance criteria, with direct implications for landing-zone selection and long-term follow-up strategy.

## Case presentation

A 60-year-old man underwent TAR-FET at another institution for acute Stanford type A aortic dissection. A frozen elephant trunk (Frozenix, 29 mm in diameter and 60 mm in length) was deployed in Zone 2 of the distal aortic arch with approximately 12% oversizing relative to the true lumen diameter. Immediately after the initial operation, rupture of the proximal anastomosis required emergent re-sternotomy, ascending aortic replacement, and left subclavian artery bypass. Thereafter, his postoperative course was uneventful, and he was followed with annual computed tomography (CT).

**Fig. 1:**
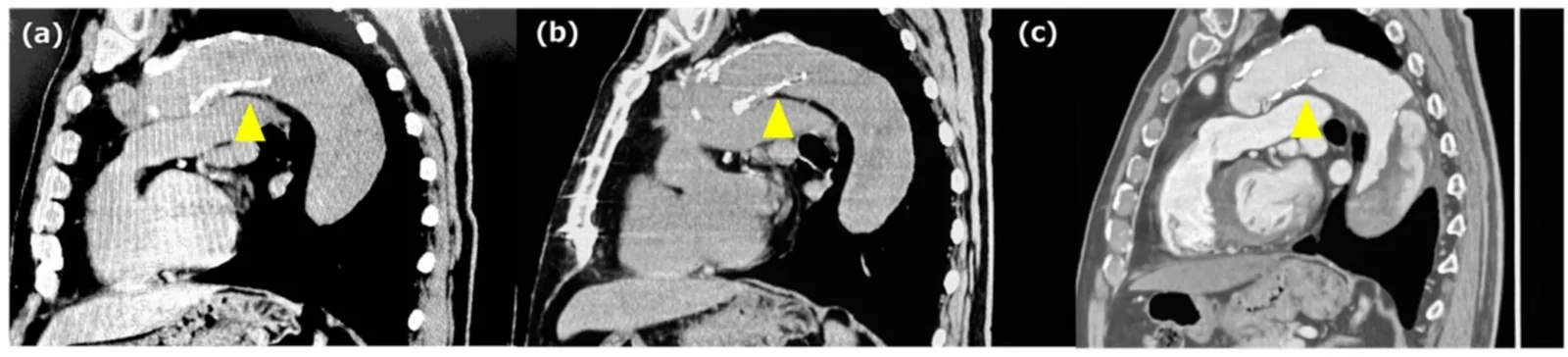
Progressive aortic arch angulation distal to the frozen elephant trunk during late follow-up. The aortic arch angle was measured on centerline-reconstructed CT as the angle between two lines drawn along the native aortic arch immediately proximal and distal to the apex of the deformity. (**a**) CT obtained 5 years after the index operation showed no apparent abnormality. (**b**) CT obtained 6 months later demonstrated newly developed mild angulation immediately distal to the FET (30°, yellow arrowhead). (**c**) CT obtained a further 6 months later showed rapid progression of the angulation to 59° (yellow arrowhead). The aortic diameter at the deformed segment remained stable at 35 mm throughout the observation period

CT obtained 5 years after the initial surgery showed no apparent abnormality. CT performed 6 months later revealed newly developed angulation immediately distal to the FET (30°). A further CT obtained 6 months later demonstrated rapid progression, with the arch angle reaching 59° (Fig. 1c, yellow arrowhead).

**Fig. 2:**
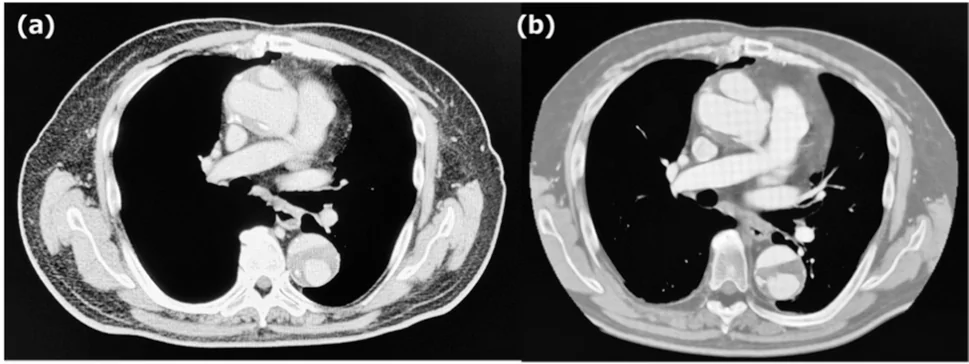
Interval enlargement of the aortic root pseudoaneurysm. Pseudoaneurysm diameter was measured as the maximal diameter on double-oblique reformatted CT images perpendicular to its long axis, at a consistent anatomical level relative to the proximal anastomosis. (**a**) The aortic root pseudoaneurysm was already identifiable at least 3 years postoperatively. (**b**) During the subsequent 6-month interval, the pseudoaneurysm enlarged by 5 mm, with a new small pericardial effusion also noted on the same study

The aortic diameter at the deformed segment remained stable at 35 mm throughout follow-up, and the patient remained asymptomatic, with normal and symmetric ankle-brachial indices (1.35 right, 1.46 left) and no symptoms of lower limb ischemia or neurological compromise. At the same time, a pseudoaneurysm of the aortic root, which had previously been followed without enlargement, showed interval growth of 5 mm during this 6-month period, together with a new small pericardial effusion (Fig. 2a and b). Given the risk of rupture and concern regarding future hemodynamic consequences, urgent surgical intervention was planned. Transthoracic echocardiography also demonstrated progression of aortic regurgitation from mild to moderate (left ventricular ejection fraction 57.7%, pressure half-time 356 ms, regurgitant volume 43.8 mL).

Because of the rapid enlargement of the pseudoaneurysm at the previously disrupted proximal anastomotic site, together with the new pericardial effusion, the risk of rupture was considered high, and urgent surgery was performed. Given the concurrent progression of the distal arch deformity, and to avoid a second, technically demanding thoracotomy in a patient who had already undergone two prior sternotomies and who was awaiting esophagectomy for a concurrently diagnosed esophageal cancer, we elected to correct the proximal pathology and extend FET coverage across the deformed segment in the same operation; TEVAR was intended to be performed electively thereafter only if required.

**Fig. 3:**
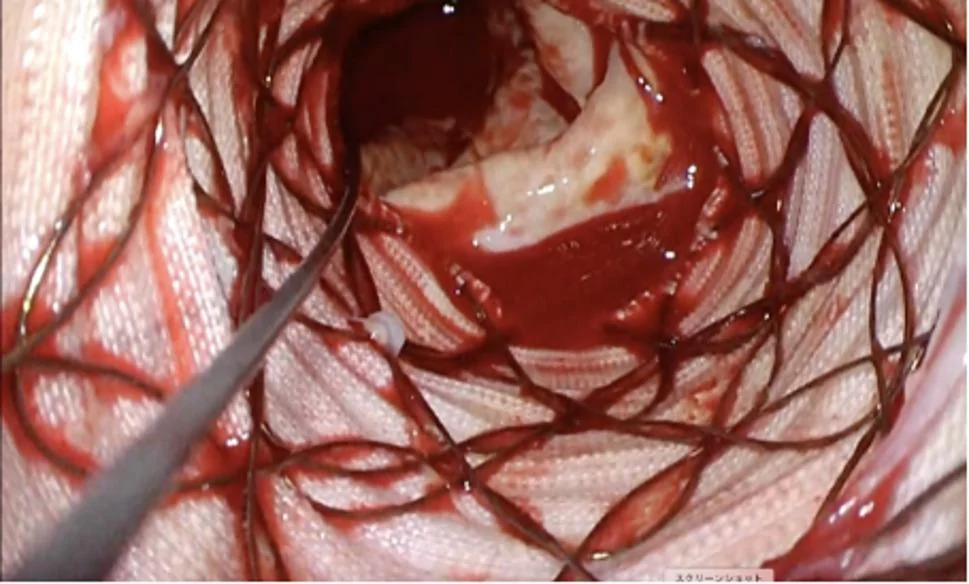
Intraoperative endoscopic findings at the tip of the frozen elephant trunk. The endoscope was advanced from the proximal (stented) segment toward the distal native aorta. Inspection after circulatory arrest demonstrated preserved intimal continuity without a discrete intimal tear. The structure visible distal to the stent tip is the deformed native aortic wall itself, which is morphologically distorted but intact. Intimal continuity was confirmed by review of the complete intraoperative endoscopic video with intermittent suction as required; the present image represents the clearest representative frame

Intraoperatively, after circulatory arrest, endoscopic inspection of the distal tip of the original FET demonstrated preserved intimal continuity and a grossly normal intimal surface throughout, without evidence of a discrete intimal tear (Fig. 3). These findings suggested that the arch deformity was unlikely to have originated from dSINE. Because of extensive adhesions around the pre-existing FET, it was preserved in situ, and a new FET (25 mm in diameter, 150 mm in length) was deployed in telescoped fashion within the existing device—sized to the existing stented lumen rather than to the native aortic wall [18]—to extend coverage beyond the apex of the arch deformity and into the straighter descending aorta. Circumferential dehiscence of the proximal anastomosis was confirmed at re-exploration. Re-total arch replacement was performed using a 26-mm graft, and a Bentall procedure using a composite graft (25-mm bioprosthetic valve conduit and 28-mm graft) was additionally required.

**Fig. 4:**
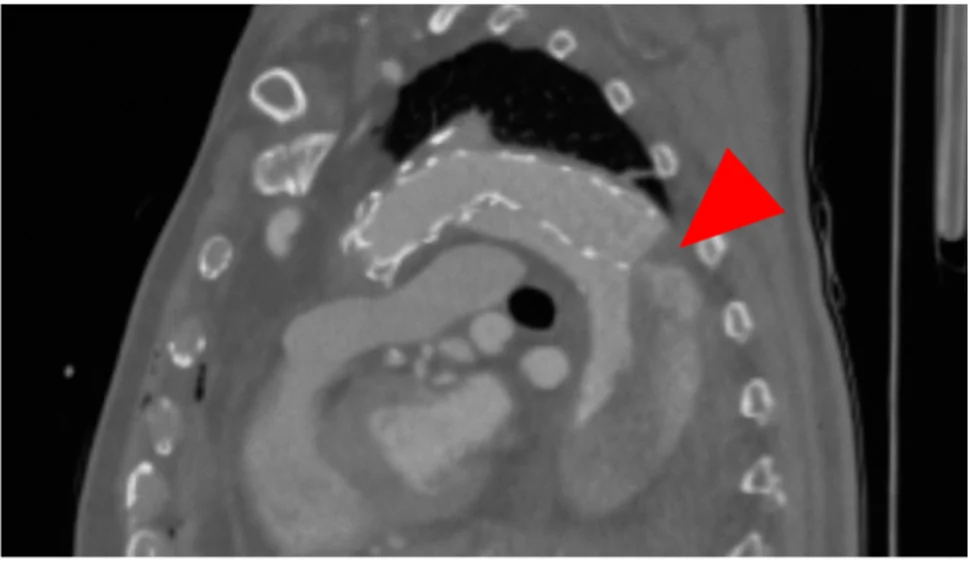
Postoperative computed tomography findings after re-total arch replacement and additional FET deployment. CT on postoperative day 1 demonstrated detachment of the distal end of the newly deployed FET from the descending aortic wall, with apparent compression of the true lumen by the false lumen (red arrow)

**Fig. 5:**
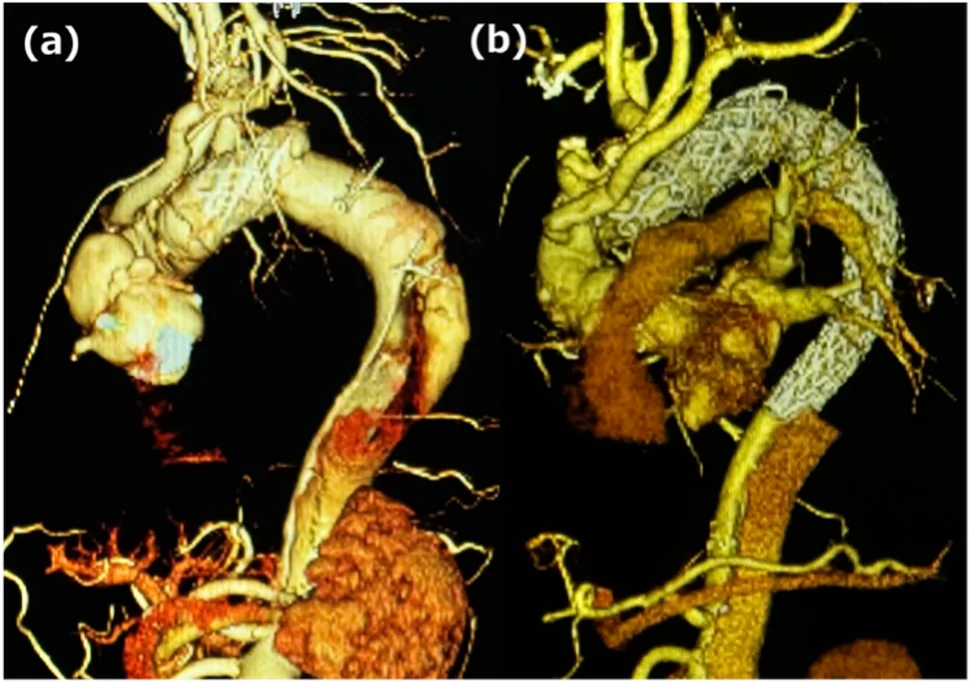
Pre- and post-TEVAR computed tomography findings. (**a**) Preoperative CT demonstrated severe angulation of the native aortic arch and a pseudoaneurysm at the proximal anastomotic site. (**b**) Postoperative CT after emergency TEVAR, performed using two overlapping stent grafts within the true lumen, demonstrated improvement of the aortic arch configuration and complete restoration of aortic morphology

The new FET migrated proximally from its intended position during this operation. No upper-to-lower extremity blood pressure gradient was apparent intraoperatively; however, on postoperative day 1, a marked gradient (30–40 mmHg) was noted in the intensive care unit and persisted despite supportive measures. CT demonstrated that the distal end of the new FET had become detached from the true lumen of the descending aorta, with apparent compression of the true lumen by the false lumen (Fig. 4a). Emergency TEVAR was therefore performed via the left common femoral artery, using two overlapping stent grafts (26 mm × 150 mm distally and 31 mm × 150 mm proximally) deployed within the true lumen, overlapping the proximal end of the new FET. Intraoperative aortography confirmed improvement of the arch configuration, exclusion of the false lumen, and absence of endoleak or access-vessel injury. The pressure gradient subsequently resolved, and postoperative CT confirmed complete restoration of aortic morphology (Fig. 5). The patient was discharged on postoperative day 15 without neurological deficit or circulatory compromise. Three months later, he underwent robot-assisted esophagectomy for the previously diagnosed esophageal adenocarcinoma, with an uneventful recovery and no recurrence at last follow-up.

## Discussion

This case is informative not primarily because any single mechanical factor is novel—spring-back force, oversizing, and short-device placement within the arch curvature have each been individually described—but because of its clinical trajectory: a deformity that was undetectable on CT at 5 years progressed to a severe, hemodynamically consequential configuration within the subsequent 6 months, all while the patient remained completely asymptomatic and showed no change in aortic diameter. We believe this trajectory, rather than any single mechanistic claim, is the principal educational message of this report.

Mechanistically, several previously described factors are likely to have converged. The original FET is, per the operative record, a single, woven-wire nitinol stent, a device class that has been reported to exert greater outward spring-back force than independent-wire devices such as the Thoraflex Hybrid prosthesis; late, spring-back-driven angle change specific to this device class has been documented in two recent Japanese cohort studies [13, 14]. The device was also sized with approximately 12% oversizing relative to the true lumen, within the 10–20% range generally recommended for the distal FET landing zone [21], and its distal end terminated within the curvature of the native arch rather than in the straighter descending aorta. Termination of a stented segment within a curved arch segment has been proposed as a mechanical risk factor through a mismatch in stiffness between the stented and non-stented aorta at this transition—the so-called aortic remodeling mismatch, or stent–flap angle—and through a short graft producing a sharp angle at the graft–native aorta interface [16, 21]. The proximal anastomosis also dehisced during this patient’s course; however, we now regard this as a largely separate process, most likely reflecting the early postoperative rupture and subsequent chronic anastomotic fragility at that site. Chronic fragility of the dissected aortic wall may have contributed modestly to both processes, but we did not identify published evidence directly linking proximal anastomotic pathology to distal arch deformity, and we have framed this association as speculative rather than causal.

Whether this deformity represents a genuinely novel late complication or simply the expected geometric consequence of the index device configuration is, in our view, not an either/or question. The eventual site of mechanical stress—where the stented segment met the curved native arch—was anatomically predictable from the index operative configuration, and indeed this predictability prompted us to perform intraoperative endoscopic screening for dSINE at the redo operation. What was not predictable from any currently established factor, however, was the timing of progression: the deformity was undetectable for 5 years and then progressed markedly within 6 months. We believe this combination—a fixed anatomical predisposition together with a time-dependent mechanical process whose tempo cannot presently be anticipated—constitutes the principal teaching point of this case, rather than either explanation in isolation.

Intraoperative endoscopic confirmation of preserved intimal continuity, without a discrete intimal tear, is in our view the strongest evidence in this case against dSINE as the underlying mechanism. dSINE has no consistently established preoperative risk factors, and its diagnosis ultimately rests on direct or imaging evidence of intimal injury rather than morphological deformity alone [15, 17, 21]. The combination of severe morphological deformity with an intact intima therefore suggests that mechanisms other than dSINE—most plausibly the device- and geometry-related factors discussed above—can independently produce clinically significant arch deformity. This distinction had direct surgical relevance in our patient: management ultimately did not differ substantially from that of dSINE with comparable hemodynamic consequences, since both the deformity and the proximal pseudoaneurysm required intervention, but the absence of an intimal tear clarified that the indication for distal extension was purely morphological rather than driven by a new entry tear.

We elected to perform re-total arch replacement with FET extension concomitantly with the Bentall procedure, rather than addressing only the proximal pathology and pursuing staged TEVAR for the distal deformity, for several reasons. With the chest already open for the proximal repair, deferring treatment of the distal deformity risked further progression, and central repair of the chronically fragile, displaced proximal aorta could plausibly have transiently worsened the distal configuration. In addition, the patient had already undergone two prior sternotomies and was awaiting esophagectomy for a concurrently diagnosed esophageal cancer; avoiding a second, technically demanding left thoracotomy in this fragile, multiply operated patient was an important consideration, particularly given the planned right-sided thoracic approach for esophagectomy and the need to preserve recurrent laryngeal nerve function and mediastinal lymph node dissection planes. The new FET was extended to 150 mm specifically to carry the distal landing zone beyond the inflection point of the arch curvature and into the straighter descending aorta, both to correct the existing deformity and to secure an adequate landing zone for anticipated future TEVAR; this length and distal positioning strategy is consistent with reports indicating that a 150-mm FET can reliably position the stent end beyond the arch curvature, in the upper-to-mid descending aorta, without increasing the risk of spinal cord ischemia [19]. In retrospect, TEVAR was required emergently on postoperative day 1 because of device migration and detachment, rather than being performed as a planned elective procedure; the rationale for the concomitant strategy—securing a stable landing zone in a single operation—nonetheless remains valid independent of this early complication.

Beyond the specific mechanism, we believe morphological deformity of the aortic arch after FET should itself be regarded as a potential risk factor warranting closer surveillance, independent of diameter and independent of dSINE. Aortic shape variation, including angulation at the level of the distal stent, has been shown to be independently associated with adverse aortic events after FET in a quantitative shape-analysis study [20]. Our case illustrates that such deformity can develop late, progress rapidly, and remain entirely silent until severe, which is consistent with the premise that morphology—and not diameter alone—carries independent prognostic information.

This report does not establish a specific morphological threshold for intervention, nor can the precise mechanism be determined from a single case. Rather, it underscores the need for long-term imaging follow-up after TAR-FET that evaluates aortic morphology in addition to diameter, even many years after the index operation, and it suggests that landing-zone selection extending beyond the arch curvature, where anatomically feasible, may reduce the risk of this complication. Future studies in larger cohorts are warranted to clarify the incidence, predictors, and clinical significance of this pattern of late arch deformity.

## Conclusion

After TAR-FET for aortic dissection, deformity of the native aortic arch may develop and progress even years after the index operation, without any change in aortic diameter or overt symptoms. This case highlights the clinical importance of long-term imaging surveillance that includes assessment of aortic morphology in addition to standard diameter measurements, and suggests that, where anatomically feasible, selecting a distal FET landing zone that extends beyond the curvature of the aortic arch into the straighter descending aorta, combined with device oversizing within the generally recommended 10–20% range, may help reduce the risk of this complication.

## Data Availability

No datasets were generated or analysed during the current study.
